# Global alternans instability and its effect on non-linear wave propagation: dynamical Wenckebach block and self terminating spiral waves

**DOI:** 10.1038/srep29397

**Published:** 2016-07-07

**Authors:** Nele Vandersickel, Arne Defauw, Peter Dawyndt, Alexander V. Panfilov

**Affiliations:** 1 Department of Physics and Astronomy, Ghent University, Krijgslaan 281, S9, 9000 Ghent, Belgium; 2 Department of Applied Mathematics, Computer Science and Statistics, Ghent University, Krijgslaan 281, S9, 9000 Ghent, Belgium; 3 Moscow Institute of Physics and Technology (State University) Dolgoprudny, Moscow Region, Russia

## Abstract

The main mechanism of formation of reentrant cardiac arrhythmias is via formation of waveblocks at heterogeneities of cardiac tissue. We report that heterogeneity and the area of waveblock can extend itself in space and can result formation of new additional sources, or termination of existing sources of arrhythmias. This effect is based on a new form of instability, which we coin as global alternans instability (GAI). GAI is closely related to the so-called (discordant) alternans instability, however its onset is determined by the global properties of the APD-restitution curve and not by its slope. The APD-restitution curve relates the duration of the cardiac pulse (APD) to the time interval between the pulses, and can easily be measured in an experimental or even clinical setting. We formulate the conditions for the onset of GAI, study its manifestation in various 1D and 2D situations and discuss its importance for the onset of cardiac arrhythmias.

The pumping function of the heart is controlled by electrical waves of excitation, which propagate through the heart and initiate cardiac contraction. Abnormal propagation of these electrical waves can result in the formation of vortices which excite the heart with a high frequency and cause a cardiac arrhythmia, called a tachycardia. In many cases, such vortices break down into complex turbulent patterns and the excitation of the heart becomes spatially asynchronous. Because of this, the effective contraction of the heart is disrupted, which results in ventricular fibrillation. Sudden cardiac death due to ventricular fibrillation is one of the largest causes of death in the industrialized world, accounting for approximately one death in ten. Therefore the mechanisms behind the initiation of vortices and the processes which can remove them from cardiac tissue are of great practical interest.

Early research showed that vortices can be formed due to regional heterogeneities in the heart. If one stimulates heterogeneous cardiac tissue with a period shorter than the refractory period of this heterogeneity, it will result in the onset of wavebreaks at this heterogeneity which can evolve into vortices^1^,^2^,^3^. The size and extent of this heterogeneity are the most important factors which underly rotor formation, and sustained vortices can be formed if the size of the heterogeneity is sufficiently large^4^,^5^.

There are many types of heterogeneities which occur due to different properties of cardiac cells in different regions of heart: e.g. the apex-base heterogeneity^6^, the transmural heterogeneity^7^, a heterogeneity around an infarction scar^8^, etc. Experimental studies of wedge preparation of the human heart also revealed local small-sized heterogeneities^9^,^10^.

In addition, the heterogeneity can occur even in homogeneous tissue as a result of action potential duration (APD) restitution, which is normally expressed in terms of the APD restitution curve. This restitution curve relates the APD and the diastolic interval (DI, the time between the end of the previous and the beginning of the new action potential) and it is normally a monotonically increasing function with saturation at large DI. Therefore, if due to some reason the DI distribution is spatially heterogeneous, for example just due to initial conditions, it will results in spatial heterogeneity in APD even if the tissue of completely homogeneous. A well known example of the onset of such a heterogeneity is the so called alternans instability, when the APD duration alternates from short to long to short etc., which was first investigated theoretically^11^,^12^ and then experimentally^13^. It was shown that it occurs if the slope of the APD restitution curve is more than one.

Such alternans instabilities obtained a lot of attention, because it was found that they result in the breakdown of a single vortex into a complex turbulent pattern of excitation^14^,^15^,^16^. These studies initiated a lot of experimental studies on the restitution properties of cardiac tissue and it was finally shown that reduction of the slope of the restitution curve prevents the onset of ventricular fibrillation^17^,^18^. In addition, a lot of new protocols and definitions of restitution relations were proposed, including dynamical restitution^19^ and the restitution portrait^20^. Additional studies indicated that the situation is more complex than originally thought: instability is not only related to the slope of the restitution curve, but also to other parameters, e.g. dispersion relation of the waves^21^,^22^. Also, two types of spatial alternans instabilities were identified: so-called concordant alternans (i.e. situation when the alternans are spatially uniform) and discordant alternans (i.e. situation when the alternans are spatially non-uniform). Discordant alternans are considered more dangerous as due to spatial heterogeneity wave blocks and reentrant patterns of excitation can occur^21^.

All studies listed above can be considered as studies of local dynamical instabilities. This means that there are some critical parameter values at which such instabilities occur. At the beginning, such an instability is usually just a small change, which grows and ultimately affects the global dynamics of the system.

The main rational of this paper is to show an important new manifestation of the APD restitution properties, which is closely related to the processes of the onset and disappearance of vortices in cardiac tissue. We will show that wave block (e.g. due to a heterogeneity) can extent into space under certain conditions, although the tissue is can be perfectly homogeneous. We will call this phenomenon a global alternans instability (GAI), as it is determined by global restitution properties of the tissue (difference of APD at two points on the restitution curve, rather than slope of the restitution curve).

In this paper, we study this instability in detail. In the first section, we will study this phenomenon in 1D and illustrate the process of growth of the wave block region, find the velocity at which this region grows and the dependency on the forcing period. Next, we propose a semi-analytical theory, and demonstrate that it can describe the observed behavior with a high accuracy. This analytical approach is similar to the one-dimensional coupled maps model used in Fox *et al*.^21^ to study alternans wave blocks which occur as a result of discordant alternans. However, in our case it is based on a quantitatively correct description of electrotonic effects developed in^23^,^24^ and evaluation of the refractory period of cardiac tissue. In the next section, we will study the GAI in 2D and show that the region of block extends itself in a similar way, and that it can result in the creation of new spiral waves or eventual removal of spirals from the tissue. Finally, show a physiological example where the GAI can be important. We end the paper with a discussion of our results and their possible consequences for the onset and dynamics of cardiac arrhythmias.

## Materials and Methods

### Model

In this paper we considered a monodomain description of cardiac tissue^25^ which has the following form:


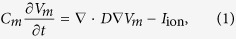


where *D* is a diffusion matrix accounting for anisotropy of cardiac tissue, *C*_*m*_ is membrane capacitance, *V*_*m*_ is transmembrane voltage, *t* is time and *I*_ion_ is the sum of ionic transmembrane currents describing the excitable behavior of individual ventricular cells. To represent human ventricular electrophysiological properties, we used the ionic TP06 model^26^,^27^. This model provides a detailed description of voltage, ionic currents, and intracellular ion concentrations for human ventricular cells. A complete list of all equations can be found in^26^,^27^. We used the default parameter settings from^27^ for epicardial cells.

### Numerical methods

For 1D and 2D simulations, the forward Euler method was applied to integrate eq. (1). A space step of Δ*x* = 0.25 mm was used in 1D, Δ*x* = 0.2 mm in 2D, and a time step of Δ*t* = 0.02 ms was used. To integrate the Hodgkin-Huxley-type equations for the gating variables of the various time-dependent currents (*m*, *h* and *j* for *I*_Na_; *r* and *s* for *I*_to_; *x*_*r*1_ and *x*_*r*2_ for *I*_Kr_; *x*_*s*_ for *I*_Ks_; *d*, *f*, *f*_2_ and *f*_Cass_ for *I*_CaL_), the Rush and Larsen scheme^28^ was used.

For a DI smaller than 32 ms, we used linear interpolation for the restitution curve, while the conduction velocity was set constant. For DI ¡ 17 ms, the APD was set equal to zero.

### Anisotropy

In all of our 2D simulations, the fibers were directed along the x-axis. In these cases the diffusion matrix was given by


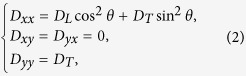


with 
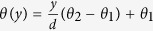
. Here *d* is the distance between epicardium and endocardium (in our simulations *d* = 16 *mm*), *θ*_1_ = −60°, *θ*_2_=60°, 
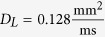
 and *D*_*T*_ = *D*_*L*_/4. For the 1D simulations, 
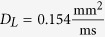
 was used.

### Ionic heterogeneities

In the 2D simulations, we have created ionic heterogeneities to induce the wave block in a realistic 2D transmural wedge. A spiral wave was induced at the left side of the medium (10 × 1.6 cm). We have then set the heterogeneity in the right side with a with of 0.48 cm, whereby we have changed the the conductance of *I*_*Ks*_ resp *I*_*K*_*r* from their standard value of *G*_*Ks*_ = 0.3923 and *G*_*Kr*_ = 0.153 *nS*/*pF* to *G*_*Ks*_ = 0.073 *nS*/*pF* and *G*_*Kr*_ = 0.048 *nS*/*pF*. This change gave a lengthening of 10 ms in the APD duration^29^. In a second simulation, we have set *G*_*Ks*_ = 0.034 *nS*/*pF* and *G*_*Kr*_ = 0.024 *nS*/*pF* to test the effect of the degree of the heterogeneity on the simulation.

## Results

### GAI in 1D

**Figure 1:**
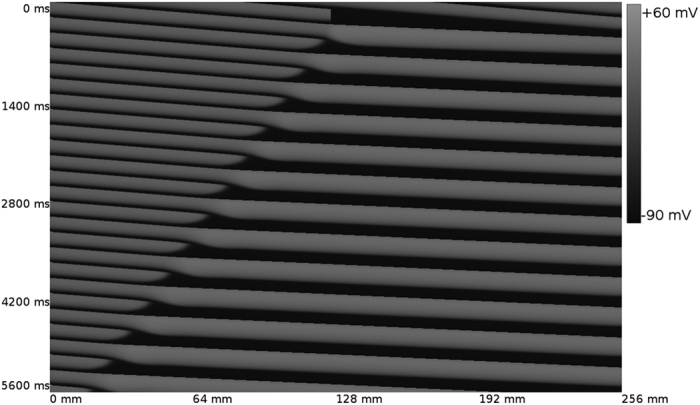
GAI in a cable of cardiac cells. The cable is paced at the left border with a period T = 220 ms. The wave block region extends itself in space. Length of the cable is 256 mm.

In Fig. 1 we show the manifestation of what we call a global alternans instability (GAI). We considered a homogeneous cable of cardiac cells, which we paced at the left border with a period T = 220 ms, corresponding to APD = 187 ms. We temporary blocked the wave propagation in the middle of the cable, so wave N cannot excite the cells to the right of this wave block location. However, this block is temporal, and wave N + 1 again excited these cells. Due to restitution effects, the duration APD_N+1_ = 278 ms is substantially longer than the normal value. As a result, the next wave N + 2 is blocked again because cells are not recovered from the action potential N + 1. In addition, if we look at Fig. 1 we observed that the point of the wave block is shifted to the left, in comparison to the previous point of block. In the same way, wave N + 3 will be able to excite the complete cable, and the process is repeated as APD_N+3_ will be longer again. So we see that in homogeneous tissue, due to initial conditions, we observe an area of wave block which extends itself in space in an alternating order. This is what we call a GAI. It is important to note that we do not observe any other alternans due to dynamical instabilities.

**Figure 2:**
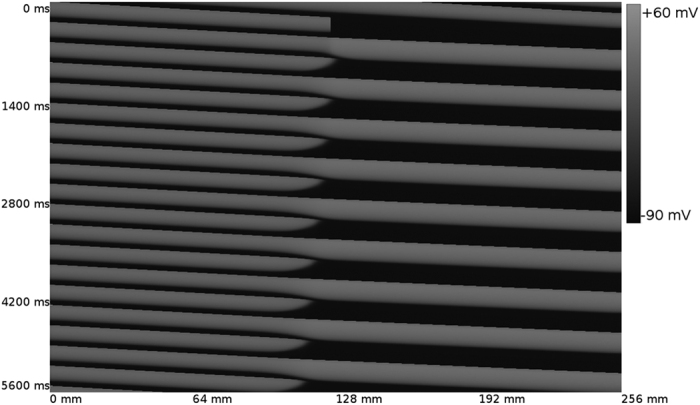
GAI in a cable of cardiac cells. The cable is paced at the left border with a period T = 290 ms. The wave block region extends itself in space. Length of the cable is 256 mm.

In Fig. 2 we show the same process but now for T = 290 ms. We observe a similar growth of the wave block region, although in this case the rate at which it grows is lower than for T = 220 ms. Again, we do not observe any other alternans in Fig. 2.

**Figure 3:**
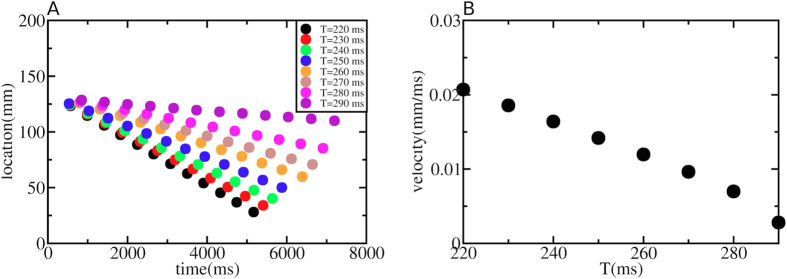
The colored dots in (**A**) show the location of the point of wave block versus time using the protocol as described in Figs 1 and 2 for different T. In (**B**) we show the velocity (in mm/ms) at which this instability extends itself in space. The velocity increases for shorter T.

Figure 3A shows the dependence of the wave block point on the period of pacing T. The colored dots show the location of block for each second wave, for a certain T. Interestingly, as was already clear from Figs 1 and 2, this shift of the wave block location is approximately constant. Next, we calculate the velocity at which the wave block region extends itself in space for different T. This is shown in Fig. 3B. We observe a linear dependency of the velocity on T. Also, we observe that there is a critical T (around 300 ms) for which this instability disappears. The reason for that is explained in the next section.

### Mechanism of GAI

#### General consideration

**Figure 4:**
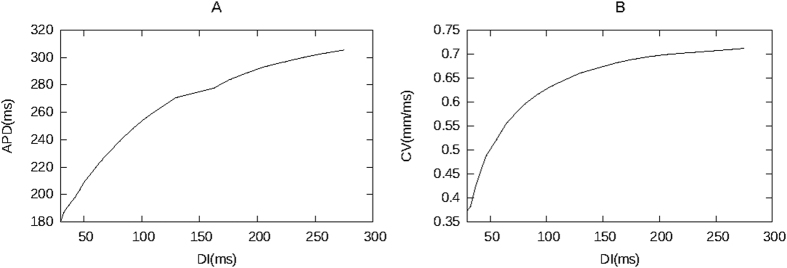
Restitution curve. DI versus APD (**A**), DI versus CV(**B**).

The critical period at which a GAI occurs can be estimated by a simple reasoning via the restitution curve (see Fig. 4). First, we note that if the cells located to the left of the point of block are stimulated with a certain period T, the cells inside the region of block are excited with a period 2 × T. Thus, if we neglect electrotonic effects, the APD for cells located outside this region is given via the restitution curve as APD_1_, which is a solution of the implicit equation APD_1_ = F_APD_(T − APD_1_), while the APD for the other cells is given by the solution of APD_2_ = F_APD_(2 × T − APD_2_). Second, we will observe a wave block if the wave arrival time is smaller than the local refractory period (RP) of the cells. So, to find when a wave block will occur, and thus a GAI, we have to relate the RP to the APD. For this, we note that in cardiac tissue APD values are closely related to RP. We find that, in our model, APD measured at 90% repolarization is approximately equal to RP, so APD ≈ RP. Finally, if we neglect conduction velocity changes, the arrival time to all points where the wave can reach is just equal to T. Combining these three remarks, we see that we will only have a GAI for those T for which:


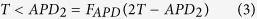


This simple formula based on the restitution curve shown in Fig. 4 gives us a critical T ≈ 310 ms, which is close to what we observed in our simulations.

#### Semi-analytical theory

We use the approach of a delay equation representation developed by Keener and Courtemanche^30^, and extended by Vinet^31^ and Fox *et al*.^21^. We will show that we can quantitatively estimate the rate at which the wave block region grows by using this analytical phenomenological approach.

For this, we assume that we deliver stimuli on the left end of the long cable with the period *T*. Let us consider a fiber on an interval [*x*_*l*_, *x*_*r*_]. For formal theoretical consideration we assume that *x*_*l*_ = −∞, *x*_*r*_ = +∞. However for numerical realization a big negative *x*_*l*_ and a large postive *x*_*r*_ will suffice. Suppose that the dynamical equilibrium is established in this system such as all points *x* of the cable have the same APD according to the restitution relation APD(*x*) = APD_0_ = *F*_APD_(*T* − APD_0_). Now suppose that we blocked a wave at some point *x* = *ξ*_0_. Then for the next wave the profile of the APD will not be uniform anymore. To find this profile we first find the profile for DI after the initial wave was manually blocked at *x* = *ξ*_0_. Let us denote the initial wave with the subscript “0” and the next wave “1”. The profile DI_0_(*x*) after the wave “0” is blocked is given by:


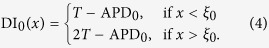


Then, for the next wave “1”, the profile of the APD should be given by: APD_1_(*x*) = *F*_APD_(DI_0_(*x*)). However, this is an oversimplification, and due to electronic effects^23^,^24^,^31^, we will have a spatially smooth transition from the points that are left of *ξ*_0_ to the points to the right of *ξ*_0_. As shown in^23^, this transition can be approximated by a convolution with a Gaussian function *G*(*x*):


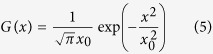


where *x*_0_ = 7.8 mm. Thus, the APD profile for the wave “1” is given by:


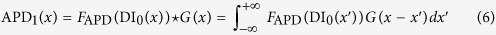


where the star 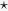 denotes the convolution. The integral in the right hand side of (6) can be evaluated in terms of the error function erf (*x*) yielding:


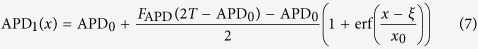


This means that the wave “2” following the wave “1” will propagate in a heterogeneous tissue with heterogeneity given by (7). If at the right boundary of the fiber (*x*_*r*_) APD_1_(*x*_*r*_) = *F*_APD_(2*T* − APD_0_) is larger than *T*, then as it follows from the criterion (3), the wave “2” will be blocked at some *x* = *ξ*_2_, which can be found from the implicit equation *APD*_1_(*ξ*_2_) = *T*.

Now, we can describe the shift of the wave block point as an iterative process using a similar analysis. For this we suppose that the wave “*n*” propagated through the whole fibre and at each point *x* of the fibre, we know *APD*_*n*_(*x*) from the previous step of the iterative procedure. Let us also assume that *APD*_*n*_(*x*_*r*_) > *T* and find the profile APD_*n*+1_(*x*). For the moment we neglect the effects of CV restitution and assume that all waves propagate with the same velocity everywhere. In this case the profile of the DI is simply given by:


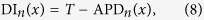


but since *APD*_*n*_(*x*_*r*_) > *T* there will be a point *x* = *ξ*_*n*+1_ where the DI_*n*_(*ξ*_*n*+1_) = 0 and wave *n* + 1 will be blocked at *x* = *ξ*_*n*+1_. Eq. (8) will be valid only for *x* ≤ *ξ*_*n*+1_ and APD_*n*+1_(*x*) will also be defined only for *x* ≤ *ξ*_*n*+1_ as:


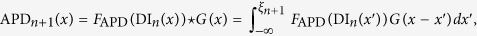


It is easy to see that for the next wave “n + 2” DI_*n*+1_(*x*), will be given by


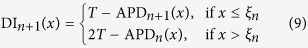


and APD_*n*+2_(*x*) for all *x* can be found from:


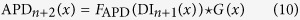


If we continue this procedure we will find the next point of wave block for the wave *n* + 3, etc and finally define the shift velocity as:


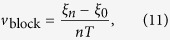


Now let us take into account the CV restitution^21^ and describe how the formulae should be adapted. If the wave velocity is no longer uniform, the interbeat interval for different points *x* is no longer a constant equal to *T* but a function of *x* which we denote as *P*(*x*). Now assume that for wave *n*, which propagates through the whole fiber without block, we know *APD*_*n*_(*x*) and DI_*n*−1_(*x*). *APD*_*n*_(*x*) is connected with DI_*n*−1_(*x*) via the equation similar to (10).

Let us consider the propagation of wave *n* + 1. The local interbeat interval between the wave *n* + 1 and *n* can be found as the difference between the time of arrival of the wave “n + 1” and the wave “n”. As the velocity of the wave *n* + 1 is determined by the DI of the previous wave CV(DI_*n*_(*x*)), we obtain:


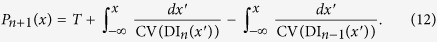


However, DI_*n*_(*x*) and *P*_*n*_(*x*) are also connected via:


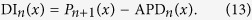


In order to solve the problems (12)–(13) it is easier to write (12) in the form of the differential equation. By differentiating the expression (12) and combining it with the formula (13), we obtain the following system:


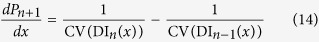



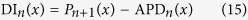



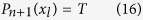


where *P*_*n*+1_ and DI_*n*_ are unknown functions of *x*, and *x*_*l*_ is location of the left boundary of the fiber where it is periodically forced with a period *T*. Numerical integration of this system starts from the left boundary and gives us values of *P*_*n*+1_ and DI_*n*_ at the successive points *x*. As in the case of constant velocity, we assume that for some *x* we will arrive to a point where DI_*n*_ is zero. This value of *x* = *ξ*_*n*+1_ will be the point of the block of the wave *n* + 1: (DI_*n*_(*ξ*_*n*+1_) = *P*_*n*+1_(*ξ*_*n*+1_) − APD_*n*_(*ξ*_*n*+1_) = 0).

Now consider propagation of the next wave *n* + 2. This wave will propagate along the whole fibre. As wave *n* + 1 propagated till the point *ξ*_*n*+1_ only, the system (14–16) will also be valid only for *x*_*l*_ ≤ *x* ≤ *ξ*_*n*+1_. In order to find an interbeat and diastolic interval for *x* > *ξ*_*n*+1_, we need to take the values of *APD* and *DI* from the previous wave *n* and add additional time *T* to *DI* as wave *n* + 2 was generated by time *T* later than wave *n* + 1. This yields the following system of equations for the wave *n* + 2:


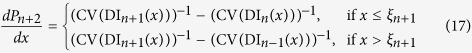



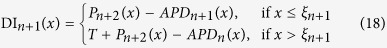



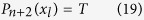


Note, that for *x* > *ξ*_*n*+1_, the interbeat interval (time between the waves *n* + 2 and *n*) will be given by *T* + *P*_*n*+2_(*x*).

The solution of this systems allows us to find DI_*n*+1_(*x*) for all *x*. Now using (10) we can find *APD*_*n*+2_(*x*) and proceed to the next iteration.

**Figure 5:**
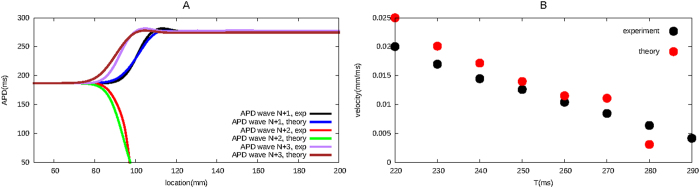
Figure A shows the APD distribution of subsequent waves in our system, obtained both via our iterative method as in numerical simulations, for a T equal to 220 ms. Black, red and green lines show APD distribution for the N + 1th, N + 2th and N + 3th wave obtained via our iterative method. Blue, purple and brown lines show APD distribution for the N + 1th, N + 2th and N + 3th wave obtained via simulations. Figure B shows the velocity at which the wave block region extends itself in space, both found via our iterative method (red dots) and as observed in numerical simulations (black dots).

Several APD profiles are shown in Fig. 5. We observe that the APD distribution obtained via our iterative method is very close to the APD distribution obtained via numerical simulations.

We tested our iterative method for different periods T, and in Fig. 5B we show the velocity at which the wave block region extends itself in space for these T, both obtained via our iterative method (red dots), as observed in the numerical simulations (green dots). We see that our theory reasonably well reproduces the numerical simulations.

#### Effect of the CV restitution

**Figure 6:**
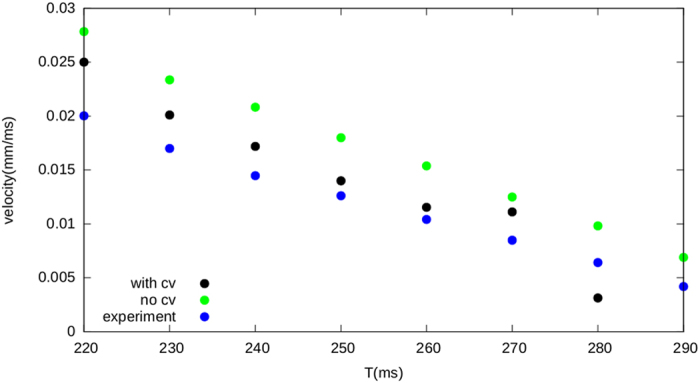
The effect of the CV restitution curve on the propagation speed of the block. Without taking the conduction velocity into account, the velocity of the block increases.

In order to explain the effect of the CV restitution, we repeated our computations without taking the CV into account and compared it to the case with the CV restitution and to the numerically obtained values. In Fig. 6, we can see that for constant conduction velocity, the velocity of the wave block increases. We can explain this results from our theory as follows. Let us consider the propagation of a wave which will be eventually blocked at some point *x*. The profile of the APD for this case is similar to the red line in Fig. 4A. where we see monotonic decrease in APD. For this transition the value of the DI for the points before the block are given by the formula (8)


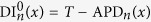


whereas if we take the CV restitution into account the formula changes as:


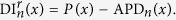


Note that we denoted the first case with the superscript “0” and the second-with “r”. The difference between these two distributions is therefore:


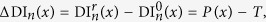


or substituting the formula (12) for *P*(*x*):


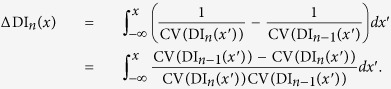


Hence, the sign of Δ DI_*n*_(*x*) is determined by the sign of the difference CV(DI_*n*−1_(*x*)) − CV(DI_*n*_(*x*)). The sign of this difference is positive. Indeed, the wave “n − 1” had a short APD and thus long DI. That means:


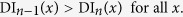


In Fig. 4B we see that larger values of DI correspond to larger values of conduction velocity, therefore:


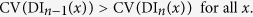


So the sign of the difference CV(DI_*n*−1_(*x*)) − CV(DI_*n*_(*x*)) is positive, meaning that the sign of Δ DI_*n*_(*x*) is also positive. Therefore, 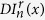 is larger than 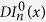 for any *x*. We define the point of the block as the point where the DI turns to zero. Since 
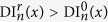
, the distibution 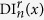 turns to zero for larger values of *x* in comparison with the distribution for 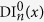. Therefore, the block propagates to the left *slower* if the CV restitution is taken into account.

#### Effect of APD restitution

**Figure 7:**
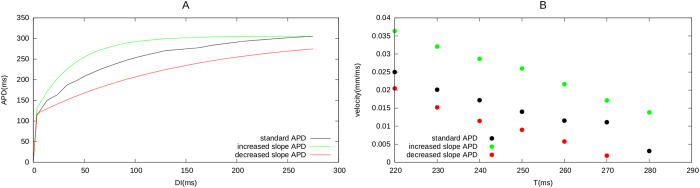
Effect of different slopes of the APD restitution curves on the velocity of the wave block.

As a final step, we considered the effect of the APD restitution curve on the propagation of the wave block. In Fig. 7, we show our results. We see that an increase in the slope of the restitution curve results in an increase of the wave block velocity. This results can also be explained as follows. An increase in the slope of the restitution curve increases the difference in *APD* for waves travelling with a period *T* and period 2*T*. Therefore, due to electrotonic effects (eq. 7) this heterogeneity spreads further in space, resulting in a more pronounced shift of the wave block.

### GAI in 2D

**Figure 8:**
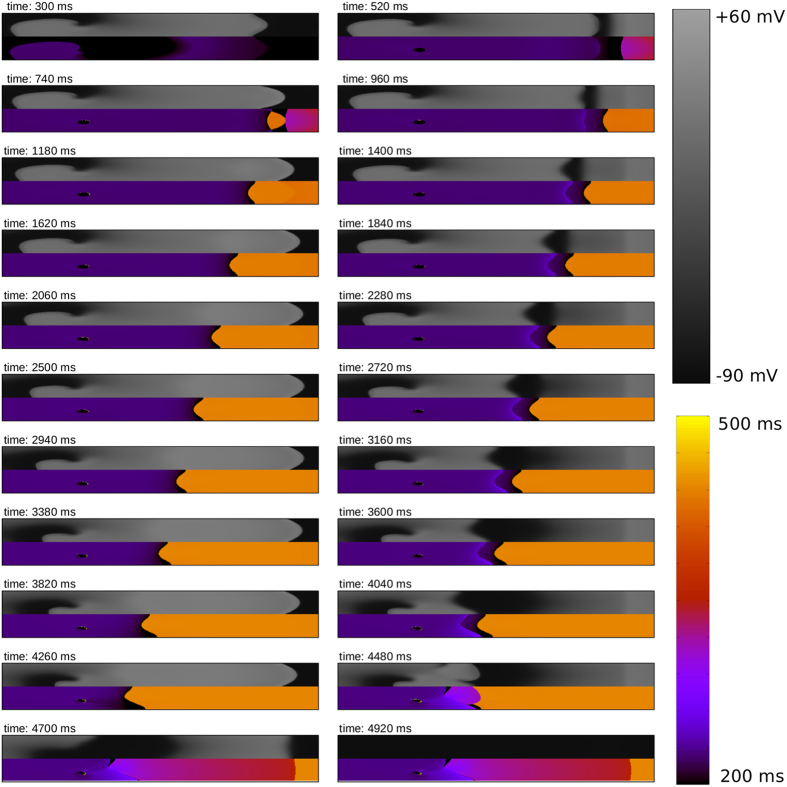
GAI in a 2D medium with rotational anisotropy. Vortex dynamics after putting a heterogeneity in the tissue at the right side of the medium. Upper panel of each timeframe shows transmembrane voltage; lower panel shows period of excitation. Size of the medium is 10 × 1.6 cm.

One question that still remains is how this initial waveblock can occur in the heart, i.e. what are the applications of this phenomenon. In order to illustrate one possible realistic effect, we have added a heterogeneity to the end of the realistic transmural wedge (including rotational anisotropy) with a thickness of just 6 mm. We initiated a rotor (spiral wave) at the left boundary and studied again the process of its interaction with the heterogeneity, as illustrated in Fig. 8 and in movie 1. As the period of the rotation of a spiral wave is shorter than the the refractory period of the heterogeneity, we first observed an expected result: each second wave is blocked at the heterogeneity, which is a well known Wenckebach block phenomenon^32^ (see also frame at t = 520 ms). However, similar as in the first 2D example, we again observed that this area of this block extended in space, closer to the spiral wave (t = 520 ms–4480 ms), and started to interact with the spiral wave which led to the termination of the spiral (t > 4700 ms).

Interestingly enough, this process does not depend on the degree of the heterogeneity. We have performed similar simulation with a larger heterogeneity (see the method section for details) and the process of interaction with the spiral wave was exactly the same: block area extension, and moreover, in both cases the spiral was terminated after exactly 4.7 s.

We note that we did many similar simulations, in which we varied the position of the initial vortex (closer, or further away from the boundaries of the medium), and each time we observed the same pattern: the wave block region approached the core of the vortex, and eventually the vortex is removed from the tissue.

We therefore conclude that a GAI is also present in 2D and has a substantial effect on vortex formation, and eventual removal of vortices. From the spatial frequency distribution, we again see that we have a clear (dynamical) Wenckebach 1:2 block, which extends itself in space.

## Discussion

In this paper we show that disturbances of wave propagation, such as temporal block of propagation, may have important effects on vortex dynamics, and can lead, for example, to vortex termination. They can also substantially affect spatial excitation patterns and result in dynamical Wenckebach blocks for wave propagation.

In our simulations such blocks occurred at a heterogeneity, or were created artificially. This is because the effect observed did not depend on the heterogeneity and we have used the most generic initial conditions. Therefore we expect the same results hold for any conditions in which a waveblock occurs. For example Sharifov *et al*.^33^ showed that parasympathetic excitation and local release of acetylcholine can result in local temporal blocks of propagation. Other mechanisms of block formation were considered by Otani^34^, mechanisms related to the source-sink mismatch by Jalife *et al*.^35^, etc. In all these cases we expect that the initial area of block under high frequency stimulation will extend itself in space and can substantially affect the wave propagation dynamics in the heart.

The fact that the block area extends itself in space is also very important as recent experimental studies showed small size heterogeneities in transmural wedges of the human heart^9^,^10^. We studied these type of heterogeneities in previous publications^29^,^36^, and we indeed found that they can create vortices but only after they had grown in space. In this paper, we illustrate that GAI is the possible mechanism of this growth of the heterogeneity.

Restitution properties of cardiac tissue were always considered as an important mechanism underlying the formation of vortices. Multiple studies^14^,^16^,^17^,^18^ showed that steep restitution can result in dynamical instabilities, possibly leading to fibrillation. Here, we show that substantial effects of restitution at the global level can also be expected according to formula (3). Thus, we show that, although steep restitution gives rise to dynamical instabilities, it is not a necessary condition: the global shape of the restitution curve also plays an important role. It would be interesting to investigate formula (3) on a patient specific restitution curve, and study if it is related to the onset of cardiac arrhythmias.

In Fox *et al*.^21^ an experimental and modeling study of spatial dynamics of discordant alternans was performed. The authors also studied the onset of conduction block under high frequency pacing. The experimental studies in Fox *et al*.^21^ were performed in 10 dog Purkinje fibers. Conduction block never developed in the absence of discordant alternans. They found that in 30% of the cases the conduction block location migrated towards the site of stimulation in a similar way as reported in our simulations. However, in 50% of the cases only paroxysmal conduction block was found, and in 20% of the cases the conduction block location was stable in space. It would be interesting to perform similar experiments in preparations where discordant alternans are not present. This can be achieved, for example, by flatterning the restitution curve^17^,^18^. We expect that wave blocks which extend in space will also be found, when the conditions given by (3) are satisfied.

The study by Fox *et al*.^21^ uses numerical simulations in a 1D ionic model and coupled iterative maps to identify the dynamical mechanism for the spatiotemporal transition to conduction block. The iterative map approach used in^21^ is similar as the one used in our study, however, there are some essential differences. In Fox *et al*. the coupled iterative maps approach was used to perform qualitative simulations for wave propagation in a 1D cable. These results were not quantitatively compared with simulations in a 1D ionic model, and used to elucidate the mechanism of the underlying phenomenon. In our study, we develop a quantitative model which is based on our human ventricular cell ionic model and use it to formulate the conditions for the onset of instability and velocity of the extension of the block area in the form of an integral equation. We also compare predictions of this analytical model with simulations and show that they are quantitatively correct.

In this paper, we only studied effects of extension of an instability (i.e. conduction blocks) in a 1D cable. In^37^ also other interesting effects were observed such as period doubling bifurcation (i.e. 1:1 → 2:2 rhythm transition) and the onset of chaos. It would be interesting to study if these regimes could be found in an ionic model for human cardiac cells. In addition if would be interesting to apply pure analytical methods used to study spatially discordant alternans, such as^38^,^39^.

## Additional Information

**How to cite this article**: Vandersickel, N. *et al*. Global alternans instability and its effect on non-linear wave propagation: dynamical Wenckebach block and self terminating spiral waves. *Sci. Rep*. **6**, 29397; doi: 10.1038/srep29397 (2016).

## Supplementary Material

Supplementary Information

Supplementary Movie S1
