# Supplementary material for: Global alternans instability and its effect on non-linear wave propagation: dynamical Wenckebach block and self terminating spiral waves
Source: Sci Rep. 2016 Jul 7;6:29397. doi: 10.1038/srep29397 (PMC4935945; doi:10.1038/srep29397)
Supplement: Supplementary Information [file srep29397-s1.pdf]

Global alternans instability and its effect on non-linear wave propagation: dynamical Wenckebach block and self terminating spiral waves.

Nele Vandersickel[1], Arne Defauw[1], Peter Dawyndt[2], Alexander V. Panfilov [1,*]

[1] **Department of Physics and Astronomy, Ghent University, Krijgslaan 281, S9, 9000 Ghent, Belgium.**

[2] **Department of Applied Mathematics, Computer Science and Statistics, Ghent University, Krijgslaan 281, S9, 9000 Ghent, Belgium.**

[*] **E-mail: alexander.panfilov@ugent.be**

Phone : +32-(0)9-2644800

Supplementary information

Movie Captions

Movie 1

GAI in a 2D medium. Vortex dynamics after putting a heterogeneity in the tissue at the right side of the medium. Movie of Figure 8.
